# (S)-crizotinib induces apoptosis in human non-small cell lung cancer cells by activating ROS independent of MTH1

**DOI:** 10.1186/s13046-017-0584-3

**Published:** 2017-09-07

**Authors:** Xuanxuan Dai, Guilong Guo, Peng Zou, Ri Cui, Weiqian Chen, Xi Chen, Changtian Yin, Wei He, Rajamanickam Vinothkumar, Fan Yang, Xiaohua Zhang, Guang Liang

**Affiliations:** 1 Chemical Biology Research Center, School of Pharmaceutical Sciences, Wenzhou Medical University, Wenzhou, Zhejiang 325035 China; 2 Department of Surgical Oncology, The First Affiliated Hospital of Wenzhou Medical University, Wenzhou, Zhejiang 325035 China; 3 Department of Interventional Radiology, The Fifth Affiliated Hospital of Wenzhou Medical University, Lishui, Zhejiang 323000 China

**Keywords:** (S)-crizotinib, Ros, ER stress, MTH1, Non-small cell lung cancer

## Abstract

**Background:**

Non–small cell lung cancer (NSCLC) accounts for approximately 80–85% of all lung cancers and is usually diagnosed at an advanced stage with poor prognosis. Targeted therapy has produced unprecedented outcomes in patients with NSCLC as a number of oncogenic drivers have been found. Crizotinib, a selective small-molecule inhibitor, has been widely used for the treatment of NSCLC patients with ALK gene rearrangements. A recent study has also shown that (S)-enantiomer of crizotinib exhibits anticancer activity by targeting the protein mutT homologue (MTH1). Since this discovery, contradictory studies have cast a doubt on MTH1 as a therapeutic target of (S)-crizotinib.

**Methods:**

NCI-H460, H1975, and A549 cells and immunodeficient mice were chosen as a model to study the (S)-crizotinib treatment. The changes induced by (S)-crizotinib treatment in cell viability, apoptosis as well as ROS, and endoplasmic reticulum stress pathway in the cells were analyzed by MTT assay, FACSCalibur, Western blotting, ROS imaging and electron microscopy.

**Results:**

Here, we report that MTH1 does not affect survival of NSCLC cells. We found that (S)-crizotinib induces lethal endoplasmic reticulum stress (ER) response in cultured NSCLC cells by increasing intracellular levels of reactive oxygen species (ROS). Blockage of ROS production markedly reversed (S)-crizotinib-induced ER stress and cell apoptosis, independent of MTH1. We confirmed these findings in NSCLC xenograft studies and showed that (S)-crizotinib-induced ER stress and cell apoptosis.

**Conclusions:**

Our results reveal a novel antitumor mechanism of (S)-crizotinib in NSCLC which involves activation of ROS-dependent ER stress apoptotic pathway and is independent of MTH1 inhibition.

**Electronic supplementary material:**

The online version of this article (doi:10.1186/s13046-017-0584-3) contains supplementary material, which is available to authorized users.

## Background

Lung cancer is the leading cause of cancer-associated deaths worldwide [1]. Non–small cell lung cancer (NSCLC) represents 80–85% of all lung cancers and is further subtyped based on defined genetic abnormalities. These genetic aberrations have also enabled the development of targeted therapeutic approaches. In particular, therapies targeting tumors carrying mutations in epidermal growth factor receptor (EGFR) or a fusion of echinoderm microtubule-associated protein-like 4 (EML4) and anaplastic lymphoma kinase (ALK) genes have been clinically successful as first-line therapies [2–5].

Crizotinib is a small-molecule inhibitor of ALK [6], c-Met [7], ROS1 [8], and is approved by US Food and Drug Administration for the treatment of advanced NSCLC with ALK rearrangements. Crizotinib has shown promise in targeting NSCLC and has clearly improved the prognosis and quality of life for patients [9]. Recent studies have highlighted the importance of crizotinib enantiomers in inhibiting different targets. ALK, c-Met, and ROS1 inhibition is attributed to (R)-crizotinib, whereas inhibition of these targets by the (S)-enantiomer of crizotinib is negligible. Interestingly, (S)-crizotinib inhibits human mutT homologue (MTH1) at nanomolar doses which is approximately 20 times more potent than the (R)-enantiomer. MTH1 is a member of the nudix phosphohydrolase superfamily of enzymes. MTH1 hydrolyzes oxidized nucleotides and prevents their incorporation into replicating DNA [10]. In colon cancer cells, (S)-crizotinib was shown to induce DNA single-strand breaks and activate DNA repair mechanisms through MTH1 inhibition. The result was suppressed tumor growth in mice, implicating MTH1 as the target of (S)-crizotinib. However, recent studies have questioned the specificity of MTH1 inhibitors including (S)-crizotinib [11–13]. What these developments have exposed is that there may be multiple mechanisms by which (S)-crizotinib mediates anti-tumor activities.

Expression of MTH1 is thought to protect cancer cells from the cytotoxic effect of high levels of reactive oxygen specifies (ROS) [14]. Although cancer cells exhibit intrinsically high levels of ROS compared to their normal counterparts, MTH1 may sanitize oxidized dNTPs by converting 8-oxo-dGTP and 2-OH-dATP into monophosphates and therefore, prevent incorporation of oxidized nucleotides into DNA [15]. Therefore, this suggests that (S)-crizotinib may allow ROS-mediated cell proliferation and survival [16, 17] while preventing the adverse effects of ROS such as promotion of cell death [18]. Whether these mechanisms are involved in NSCLC in not known. In this study, we have tested the hypothesis that (S)-crizotinib inhibits NSCLC growth by a MTH1-independent mechanism. In culture studies and in tumor xenografts produced in mice, we found that (S)-crizotinib induces apoptosis in NSCLC cells through the elevation of ROS and subsequent activation of the ER stress pathway. We also show that these activities are independent of MTH1.

## Methods

### Reagents

(S)-crizotinib (Selleck Chemical, Shanghai, China) was reconstituted in dimethyl sulfoxide (DMSO) and stored at −20 °C. The antioxidants N-acetyl-L-cysteine (NAC), glutathione (GSH), and superoxide dismutase (SOD) were purchased from Beyotime Biotech (Nantong, China). Antibodies against B cell lymphoma 2 (Bcl-2), Bcl-2 associated protein x (Bax), Ki67, GAPDH, and horseradish peroxidase-conjugated secondary antibodies were purchased from Santa Cruz Biotechnology (Santa Cruz, CA). Antibodies against activating transcription factor 4 (ATF4), phosphorylated and total eukaryotic initiation factor 2α (eIF2α), and CCAAT/−enhancer-binding protein homologous protein (CHOP) were purchased from Cell Signaling Technology (Danvers, MA). FITC Annexin V apoptosis Detection Kit I and Propidium Iodide (PI) were purchased from BD Pharmingen (Franklin Lakes, NJ).

### Cells and cell culture

Human NSCLC cell lines (NCI-H460, H1975, and A549) were purchased from the Institute of Biochemistry and Cell Biology, Chinese Academy of Sciences. NCI-H460 and H1975 cells were cultured in RPMI-1640 medium (Gibco, Eggenstein, Germany), whereas A549 were grown in F12 K medium (Gibco, Eggenstein, Germany). All media formulations were supplemented with 10% heat-inactivated fetal bovine serum (Gibco, Eggenstein, Germany).

### Cell viability assay

To measure viability, cells were first seeded at a density of 8 × 10^3^ per well in 96-well plates and cultured for 24 h in normal growth media to allow attachment. Cells were then treated with (S)-crizotinib. Initial studies were performed with 24 h culture of cells with final concentrations of (S)-crizotinib at 0.625, 1.25, 2.5, 5, 10, 20,40,60,and 80 μM. All other studies were performed with concentrations and time of exposure as indicated. Following treatments, media was replaced with fresh media containing 0.5 mg/mL MTT reagent. Cells were cultured for an additional 4 h. DMSO (100 μL) was added to dissolve the formazan product and absorbance was measured at 490 nm using RT 6000 microplate reader. The percent inhibition rate was calculated as [1 − (treated/control)] × 100. The IC50 values were obtained using the Logit method.

### Measurement of reactive oxygen species generation in cells

Cellular ROS generation was measured by flow cytometry. Briefly, 5 × 10^5^ cells were plated in 6-well culture dishes and allowed to attach overnight. Cells were then treated with (S)-crizotinib at concentrations and times indicated. Cells were stained with 10 μM DCFH-DA or 5 μM DAF-FM-DA (Beyotime Biotech, Nantong, China) at 37 °C for 30 min. Cells were harvested and then washed three times with ice-cold PBS and fluorescence was measured by flow cytometry (FACSCalibur, BD Biosciences, CA). In some experiments, cells were pretreated with 5 mM NAC, 5 mM GSH, or 300 U/mL SOD for 1 h. In all experiments, 8000 viable cells were analyzed.

### Cell apoptosis analysis

NCI-H460, H1975 and A549 cells were plated at 2.5 × 10^5^ cells/well in 6-well culture dishes for 24 h and treated with (S)-crizotinib (10, 20 or 30 μM) for 24 h. Cells were then collected, washed with ice-cold PBS, and evaluated for apoptosis by double staining with FITC conjugated Annexin V and Propidium Iodide (PI). Data were collected and analyzed using FACSCalibur flow cytometer.

### Western blot analysis

Cells or tumor tissues were homogenized in protein lysis buffer, and debris was removed by centrifugation for 10 min at 4 °C. Protein concentrations in all samples were determined by using Bradford protein assay (Bio-Rad, Hercules, CA). Protein samples were separated using 6–12% sodium dodecyl sulfate-polyacrylamide gels and transferred to PVDF membranes. Five percent nonfat milk was used to block nonspecific binding for 1 h at room temperature. Blots were then probed with specific primary antibodies. Horseradish peroxidase-conjugated secondary antibodies and ECL kit (Bio-Rad, Hercules, CA) were used for detection.

### siRNA knockdown experiments

The siRNA duplexes used in this study were purchased from Invitrogen (Carlsbad, CA, USA). CHOP (NM_004083, sense 5′-GAGCUCUGAUUGACCGAAUGGUGAA-3′), and MTH1 (NCBI accession: sense 5′-CGACGACAGCUACUGGUUUTT-3′) siRNA were custom-designed. Negative Universal Control (Invitrogen) was used as the control. NCI-H460 cells were seeded at 3 × 10^5^/well into 6-well plates and cultured for 24 h. Cells were then transfected with siRNA (100 nM final concentration) or control siRNA by lipofectamine 2000 (Invitrogen). Forty-eight hours after transfection, levels of CHOP and MTH1 proteins were detected by Western blot.

### ER electron microscopy

NCI-H460 cells were exposed to vehicle control (DMSO) or (S)-crizotinib (30 μM), with or without 5 mM NAC pretreatment for 1 h. Cells were then fixed with 2.5% glutaraldehyde overnight at 4 °C, post-fixed in 1% OsO4 at room temperature for 60 min, stained with 1% uranylacetate, and embedded in epon. Areas containing cells were block-mounted and cut into 70 nm sections and examined with an electron microscope (H-7500, Hitachi, Ibaraki, Japan).

### In vivo xenograft model

Animal studies were reported in compliance with the ARRIVE guidelines [19, 20]. All animal experiments complied with the Wenzhou Medical University’s Policy on the Care and Use of Laboratory Animals. Protocols for animal studies were approved by the Wenzhou Medical College Animal Policy and Welfare Committee (Approved documents: 2016/APWC/0046) and followed the National Institutes of Health guide for the care and use of Laboratory animals (NIH Publications No. 8023, revised 1978). Five-week-old, athymic BALB/c nu/nu female mice (17-19 g, totally *n* = 15) were purchased from Vital River Laboratories (Beijing, China). Mice were housed at a constant room temperature with a 12 h:12 h light/dark cycle and fed a standard rodent diet and given water ad lib. The mice were divided into three experimental groups on randomization and blinding. NCI-H460 cells were injected subcutaneously into the right flank of mice at 1 × 10^7^ cells in 150 μL of PBS. When tumors reached a volume of 50–100 mm^3^ (approximately 6 d), mice were treated by intraperitoneal injections of (S)-crizotinib once daily (7.5, or 15 mg/kg) for 10 days. The tumor volumes were determined by measuring length (l) and width (w) and calculating volume (V = 0.5 × l × w^2^) at the indicated time points. At the end of treatment, the animals were sacrificed after being anesthetized by i.p. injection with pentobarbital sodium (50 mg·kg^−1^) and the tumors were isolated by surgery in a room separated from the other mice, and then the tumors were removed and weighed for use in histology and proteins expression studies.

### ROS determination in tumor tissues

In order to analyze ROS levels in tumor tissue, we stained tissue sections with 10 μM dihydroethdium (DHE) and 50 μM DCFH-DA for 30 min. Images were captured under a fluorescence microscope (Nikon, Japan).

### Malondialdehyde (MDA) assay

Tumors samples from mice were homogenized and sonicated. Tissue lysates were then centrifuged at 12,000 xg for 10 min at 4 °C to collect the supernatant. Total protein content was determined by the Bradford assay. Malondialdehyde (MDA) levels were measured by using Lipid Peroxidation MDA assay kit (Beyotime Institute of Biotechnology).

### Immunohistochemistry

Tumor tissues were fixed in 10% formalin at room temperature, processed and embedded in paraffin. Parraffin-embedded tissues were sectioned at 5 μm thickness. Tissue sections were rehydrated and incubated with primarily antibodies at 4 °C overnight. Conjugated secondary antibodies and diaminobenzidine (DAB) were used for detection. Images were captured using Image-Pro Plus 6.0 (Media Cybernetics, Inc., Bethesda, MD). Liver, kidney, and heart tissues from mice were subjected to routine hematoxylin and eosin (H&E) staining.

### Statistical analysis

All experiments are randomized and blinded. The data and statistical analysis in this study complies with the recommendations on experimental design and analysis in pharmacology [21]. All data are shown as mean ± SEM. Statistical analysis was performed with GraphPad Prism 5.0 software (San Diego, CA, USA). In accordance with Journal policy, statistical analysis was performed only when a minimum of *n* = 5 independent samples was acquired. One-way ANOVA followed by Dunnett’s post hoc test was used for comparing more than two groups of data and one-way ANOVA, non-parametric Kruskal–Wallis test, followed by Dunn’s post hoc test was used when comparing multiple independent groups. All other results were analysed using unpaired t-test for comparison between two groups. Values of at least *P* < 0.05 were considered statistically significant. Post-tests were run only if F achieved *P* < 0.05 and there was no significant variance in homogeneity.

## Results

### (S)-crizotinib increases ROS production and induces apoptosis in NSCLC cells independently of MTH1

**Fig. 1:**
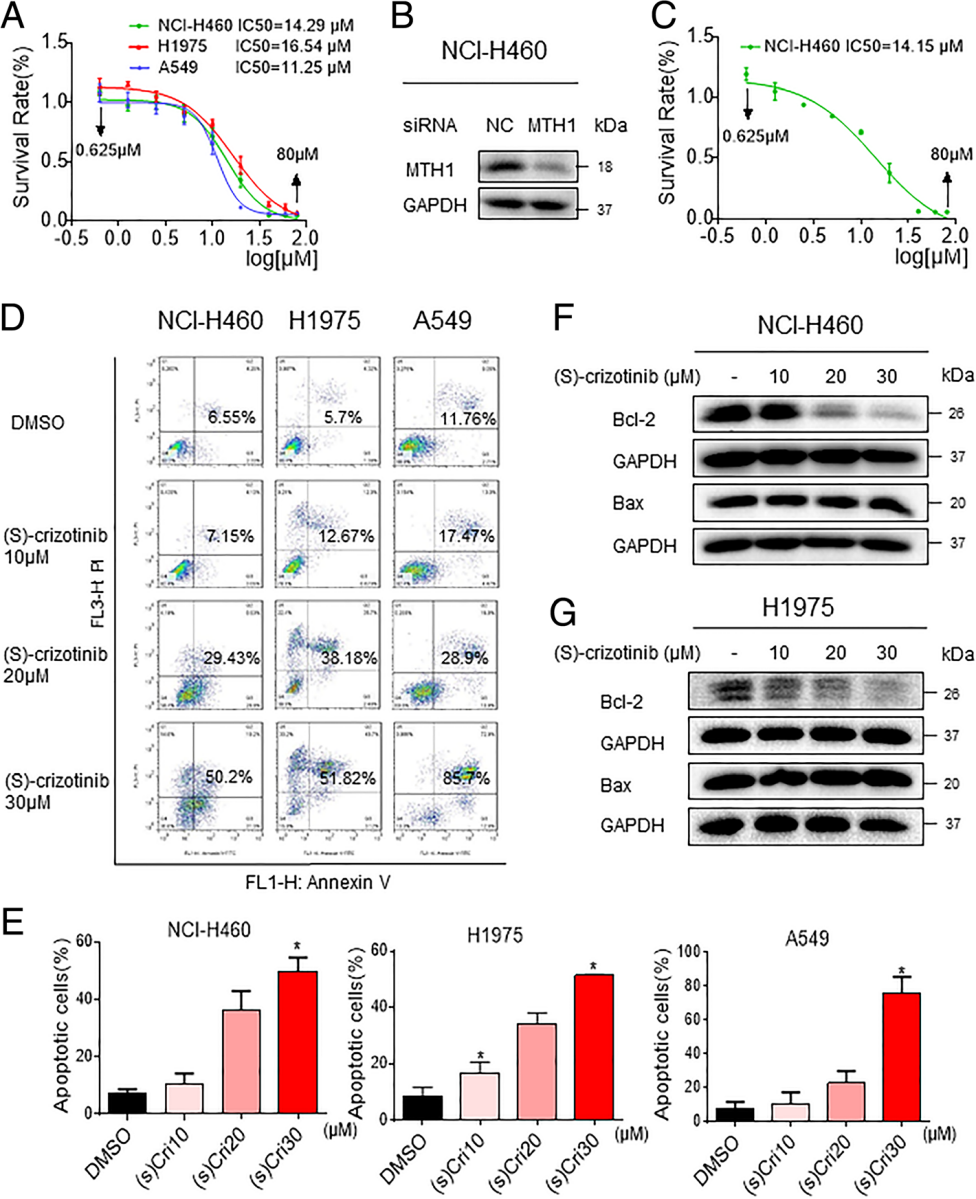
(S)-crizotinib induces NSCLC cell apoptosis independently of its effect on MTH1. **a** The effects of (S)-crizotinib on the viability of human NSCLC cells. NCI-H460, H1975, and A549 cells were challenged with increasing doses of (S)-crizotinib (0.625–80 μM) for 24 h. Cell viability was determined by MTT assay and the IC_50_ values (24 h) were calculated. **b** NCI-H460 cells transfected with siRNA against MTH1 and whole cell lysates were used to assess MTH1 protein levels. GAPDH was used as internal control. **c** Effect of MTH1 knockdown on the viability of NCI-H460 cells. **d** Induction of apoptosis in NSCLC cells was determined by annexin V/PI staining of cells following treatment with (S)-crizotinib (10, 20 or 30 μM) for 24 h. **e** Quantification of annexin V/PI staining showing the percentage of apoptotic cells following (S)-crizotinib treatment [*P < 0.05 compared to DMSO control]. NCI-H460 **f** and H1975 **g** cells were treated with (S)-crizotinib for 24 h. Cell lysates were then subjected to assessment of apoptosis-related proteins by western blot. GAPDH was used as internal control. Data representative of 5 independent experiments

To investigate the effect of (S)-crizotinib on the viability of human NSCLC cells, NCI-H460, H1975 and A549 cells were treated with (S)-crizotinib for 24 h and the number of live cells were measured by the MTT assay. As shown in Fig. 1a, (S)-crizotinib decreased the viability of NCI-H460, H1975 and A549 cells with IC_50_ values of 14.29, 16.54 and 11.25 μM, respectively. We then knocked down the expression of MTH1 in NCI-H460 cells using siRNA (Fig 1b) to determine whether (S)-crizotinib regulated viability through MTH1. If (S)-crizotinib did mediate its effects through MTH1, then MTH1 knockdown would be expected to mimic (S)-crizotinib. Interestingly, siRNA knockdown of MTH1 did not alter the viability either alone or following (S)-crizotinib treatment (Fig 1c). The IC_50_ values were almost identical with or without siRNA knockdown of MTH1 suggesting that the effect of (S)-crizotinib on NSCLC viability is independent of MTH1.

We next determined whether reduced viability of cells following (S)-crizotinib was due to apoptosis. Staining of cells with annexin V/Propidium Iodide (PI) showed induction of cellular apoptosis in all three NSCLC lines after (S)-crizotinib exposure (Fig. 1d and e). To confirm this finding, we examined the levels of apoptosis-related proteins in NCI-H460 and H1975. Our results show that (S)-crizotinib decreased B cell lymphoma 2 (Bcl-2) (Fig 1f). In addition, Bcl-2 associated protein x (Bax) was either unaltered (H460 cells) or showed an increase (H1975 cells). In either case, decreased Bcl-2: Bax ratio signified induction of apoptosis in cells after (S)-crizotinib treatment.

### ROS generation is upstream of (S)-crizotinib-induced apoptosis

**Fig. 2:**
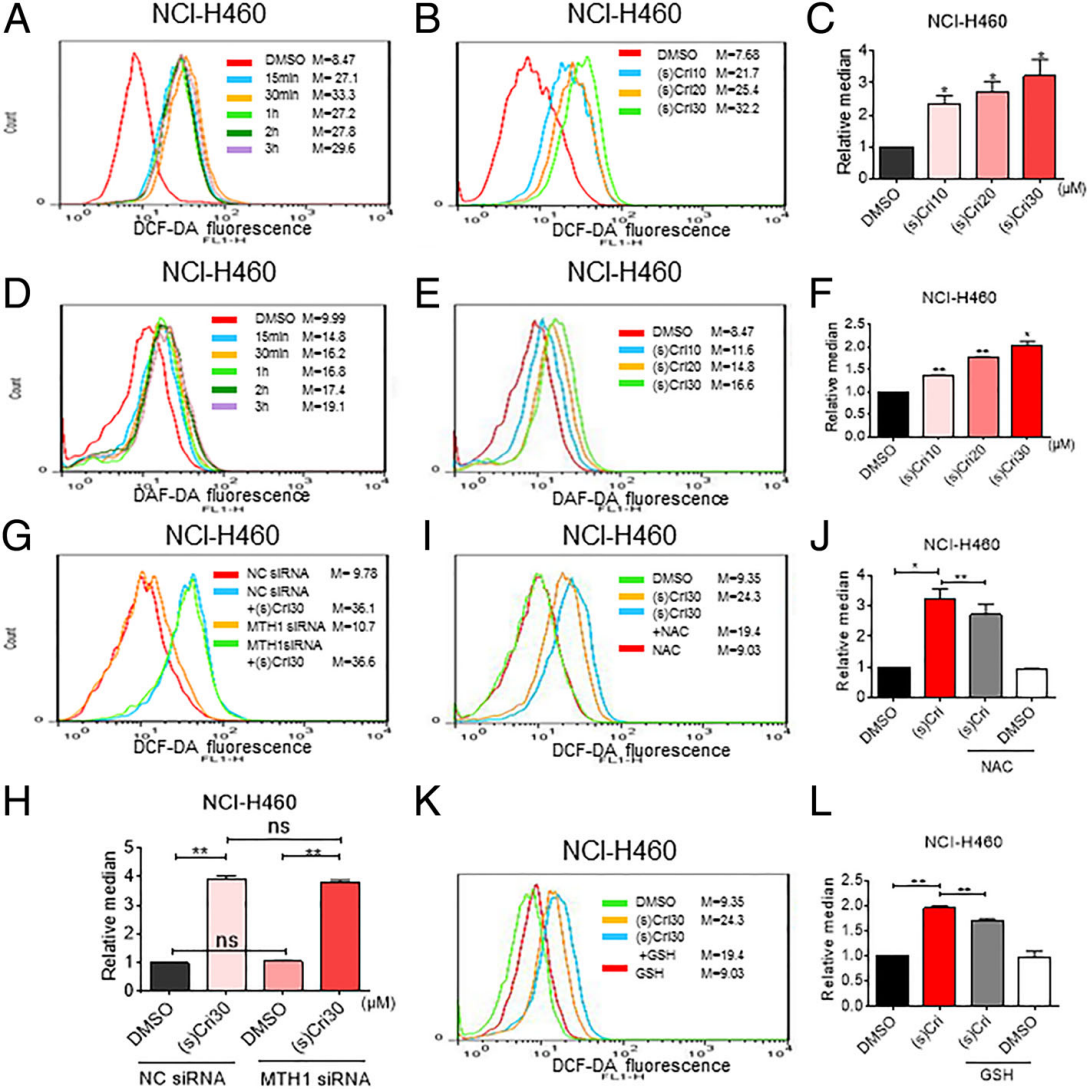
(S)-crizotinib induces ROS in NSCLC cells independent of MTH1. **a**-**c** Intracellular ROS generation as assessed by DCF fluorescence in cells following exposure to (S)-crizotinib for different time periods (a, 30 μM) and different concentrations (**b**). Quantification of data in (**b**) is shown in panel (**c**) [*P < 0.05 compared to DMSO control]. **d**-**f** Levels of nitric oxide production in NSCLC cells as measured by DAF-FM fluorescence. Cells were treated as outlined in **a**-**c**. Quantification of data in (**e**) is shown in panel (f) [*P < 0.05, **P < 0.01 compared to DMSO control]. **g**-**h** Effect of MTH1 knockdown on ROS production (DCF fluorescence) in NCI-H460 cells [**P < 0.01, ns = no significance]. **i**-**l** NCI-H460 cells were treated with 30 μM (S)-crizotinib with or without 1 h pretreatment with 5 mM NAC (**i**) or 5 mM GSH (**k**) The relative mean fluorescence intensity is presented in (**j**) and (**l**) [*P < 0.05 and **P < 0.01]

Previous studies have reported that ROS generation in NSCLC is able to trigger cell apoptosis [22, 23]. Crizotinib also increases oxidative stress in ovarian cancer cells [24] and alveolar cells [25] Therefore, we determined whether apoptosis and reduced viability in NSCLC cells following (S)-crizotinib treatment involves changes in intracellular ROS levels. We measured ROS generation in cells through 2′,7′–dichlorofluorescin diacetate (DCFH-DA) staining. DCFH-DA undergoes hydrolysis and then ROS-mediated oxidation to a fluorescent state, providing a measure of ROS levels in cells. We also measured nitric oxide levels as an additional readout of oxidative stress [26] through 4-amino-5-methylamino-2′,7′-difluorofluorescein diacetate (DAF-DA). As shown in Fig. 2a-f, treatment of NCI-H460 cells with (S)-crizotinib caused a rapid, dose-dependent increase in ROS levels (DCF and DAF levels). The same result was obtained in A549 and H1975 cells (Additional file 1: Figure S1a). We then assayed cells following MTH1 siRNA transfection to determine whether MTH1 deficits mimic (S)-crizotinib treatment. We found that MTH1 knockdown did not alter ROS levels with or without (S)-crizotinib treatment (Fig. 2g-h). However, pretreatment of cells with N-acetyl cysteine (ROS scavenger; NAC, 5 mM) and glutathione (GSH, 5 mM) significantly reduced (S)-crizotinib-induced increases in DCF fluorescence as expected (Fig. 2I-L, and Additional file 1: Figure S1b, S1c). Interestingly, pretreatment with superoxide dismutase (SOD) failed to reduce DCF fluorescence caused by the exposure of cells to (S)-crizotinib (Additional file 1: Figure S1d). These results indicate that (S)-crizotinib-induced ROS generation is independent of MTH1 alteration. The results also point to hydrogen peroxide and nitric oxide, but not superoxide anion, to be involved.

**Fig. 3:**
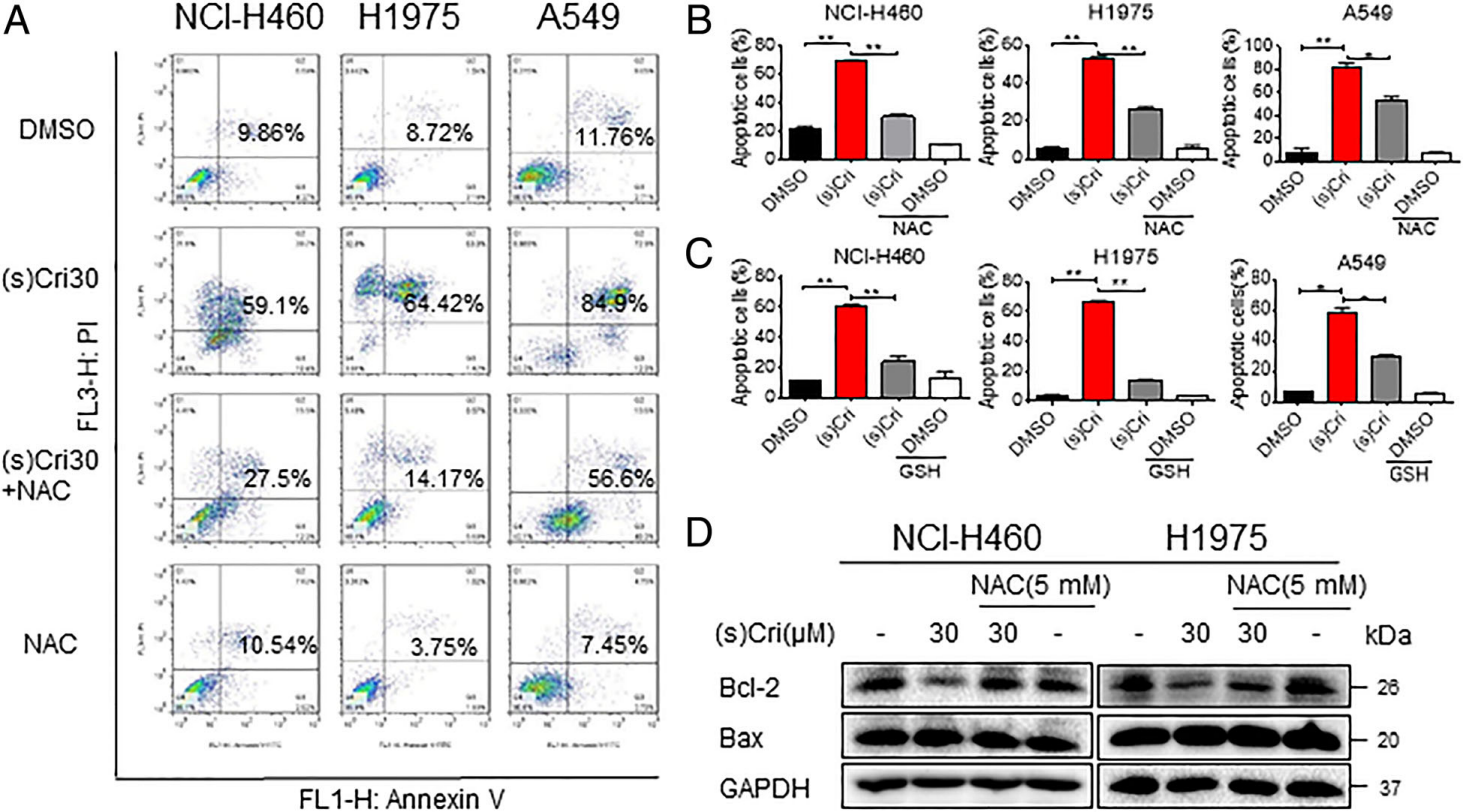
Blocking ROS abolishes the cytotoxicity of (S)-crizotinib. a-c NSCLC cells were pre-treated with 5 mM NAC (**a**-**b**) or 5 mM GSH (**c**) for 1 h before exposure to 30 μM (S)-crizotinib for 24 h. Extent of cell apoptosis was determined by annexin-V/PI staining. **b** and **c** show quantification of flow cytometry data [*P < 0.05 and **P < 0.01]. **d** Apoptosis-related proteins were measured in NCI-H460 and H1975 cells following treatment as outlined in (**a**) and (**b**). GAPDH was used as internal control

We next wanted to know if (S)-crizotinib-induced ROS was mediating cell apoptosis. We stained cells with Annexin V/PI following exposure to (S)-crizotinib with or without pretreatment with NAC, GSH, and SOD. Based on our ROS level findings above, we expected NAC and GSH to abolish (S)-crizotinib-induced cell apoptosis but not SOD. Indeed, pretreatment of cells with NAC and GSH significantly inhibited apoptosis induced by (S)-crizotinib (Fig. 3a-c). As expected, pretreatment with SOD failed to protect cells against (S)-crizotinib-induced cell apoptosis (Additional file 1: Figure S2). These changes paralleled levels of Bcl-2 protein in cells (Fig. 3d).

### ER stress pathway is activated during (S)-crizotinib-induced apoptosis

**Fig. 4:**
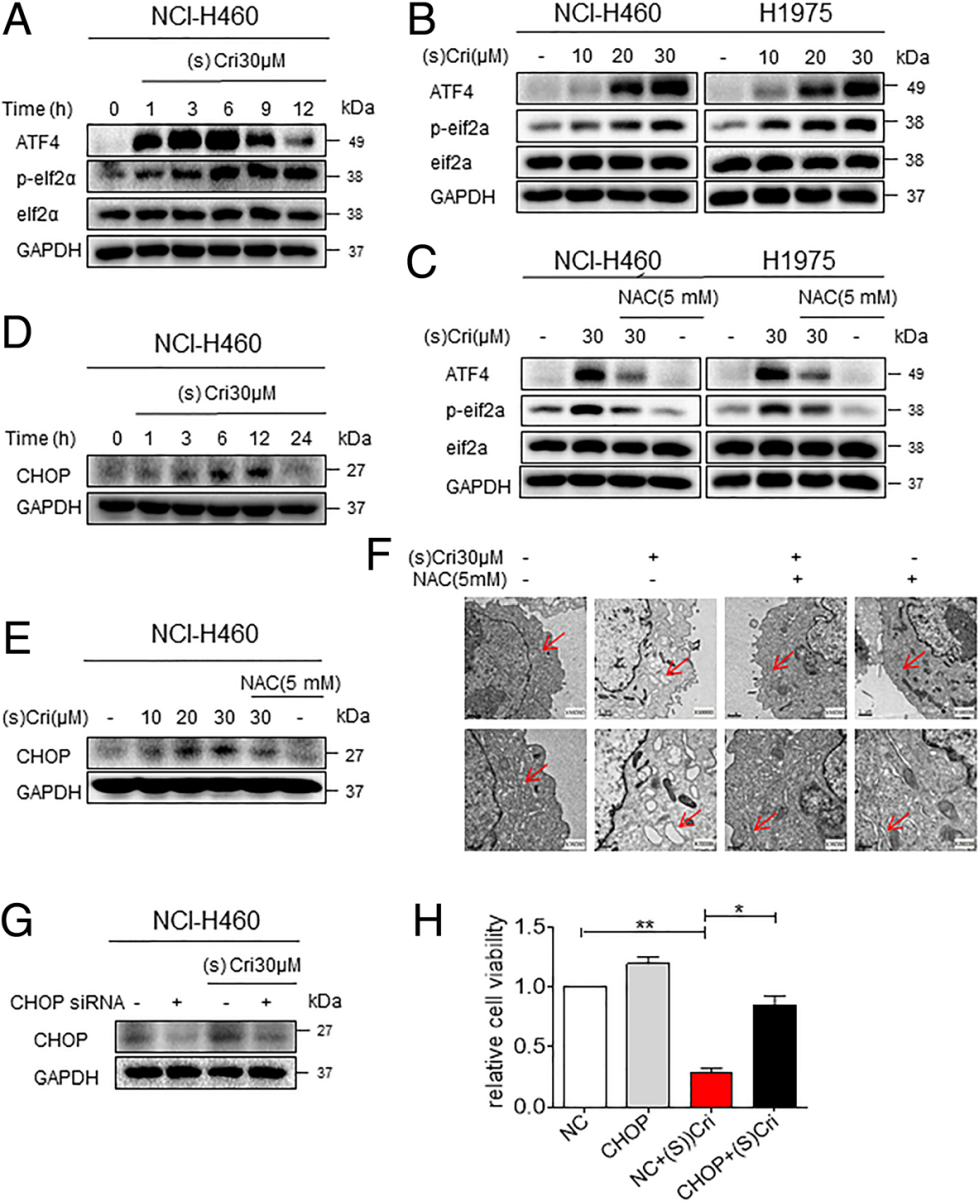
ER stress is involved in (S)-crizotinib-induced NSCLC cell apoptosis. **a** NCI-H460 cells were treated with 30 μM (S)-crizotinib for the indicated times and protein levels of ATF4, phosphorylated eIF2α, and CHOP were determined by western blot. GAPDH was used as loading control. **b** NCI-H460 and H1975 cells were treated with (S)-crizotinib at different concentrations for 6 h. Cell lysates were then subjected to western blotting for ATF4 and p-eIF2a levels. **c** NCI-H460 and H1975 cells were pretreated with 5 mM NAC for 1 h before exposure to (S)-crizotinib for 6 h. ATF4, p-eIF2α levels were detected by western blot. **d**-**e** Levels of CHOP in NCI-H460 cells following treatment with 30 μM (S)-crizotinib for different time periods. The exposure to (S)-crizotinib was carried out with or without pretreatment of cells with 5 mM NAC for 1 h (**e**). **f** Effect of (S)-crizotinib on the morphology of endoplasmic reticulum in NCI-H460 cells. Cells were treated with 30 μM (S)-crizotinib before examination by an electron microscope (×10,000 or ×20,000). Presentative micrographs from three independent experiments are shown. **g**-**h** NCI-H460 cells were transfected with siRNA against CHOP or control siRNA. Cells were then treated with 30 μM (S)-crizotinib. GAPDH was used as internal control for western blot analysis (**g**). Relative cell viability was determined by counting viable cells under a microscope (**h**) [*P < 0.05 and **P < 0.01]

Increased ROS levels and perturbation in the intracellular redox status increase the levels of unfolded proteins in the ER and induce ER stress response [27]. Unfolded protein response induces protein kinase R (PKR)-like endoplasmic reticulum kinase (PERK)-mediated phosphorylation of eukaryotic initiation factor-2α (eIF2α). Phosphorylated-eIF2α the blocks protein translation but allows preferential translation of activating transcription factor 4 (ATF4). ATF4 is a key transcription factor in the ER stress pathway and mediates the induction of the pro-death transcriptional regulator CCAAT/−enhancer-binding protein homologous protein (CHOP). Thus, we examined the expressions of these ER stress-related proteins in NSCLC cells following (S)-crizotinib treatment. Our results show that (S)-crizotinib rapidly induces the phosphorylation of eIF2α and protein levels of ATF4 in NCI-H460 cells (Fig. 4a). These inductions were associated with increased CHOP protein levels (Fig. 4d). In addition to rapid activation of the ER-stress pathway, we noted that (S)-crizotinib activated the pathway in a dose-dependent manner (Fig. 4b and e). Importantly, pre-treatment of cells with NAC prevented (S)-crizotinib-induced levels of these ER stress proteins (Fig. 4c and e).

In addition to induction of proteins associated with the ER stress pathway, we noted perturbations in the morphology of ER through electron microscopy. Compared to control (DMSO-treated) NCI-H460 cells, the ER in cells exposed to (S)-crizotinib exhibited swelling (Fig 4f). These changes were not observed in cells pretreated with NAC. Both, morphological change and induction of ER stress proteins, suggest that ROS-mediated ER stress pathway causes apoptosis in NSCLC cells. Since CHOP is the downstream effector protein in ER stress-induced apoptosis, we knocked down the expression of CHOP (Fig 4g) and then exposed the cells to (S)-crizotinib to measure viability. As shown in Fig. 4h, knockdown of CHOP normalized (S)-crizotinib-induced changes in cell viability. Together, these data demonstrate that (S)-crizotinib-induced apoptosis is, at least in part, mediated through the activation of the ER stress pathway.

### (S)-crizotinib inhibits NCI- H460 xenograft tumor growth in vivo

**Fig. 5:**
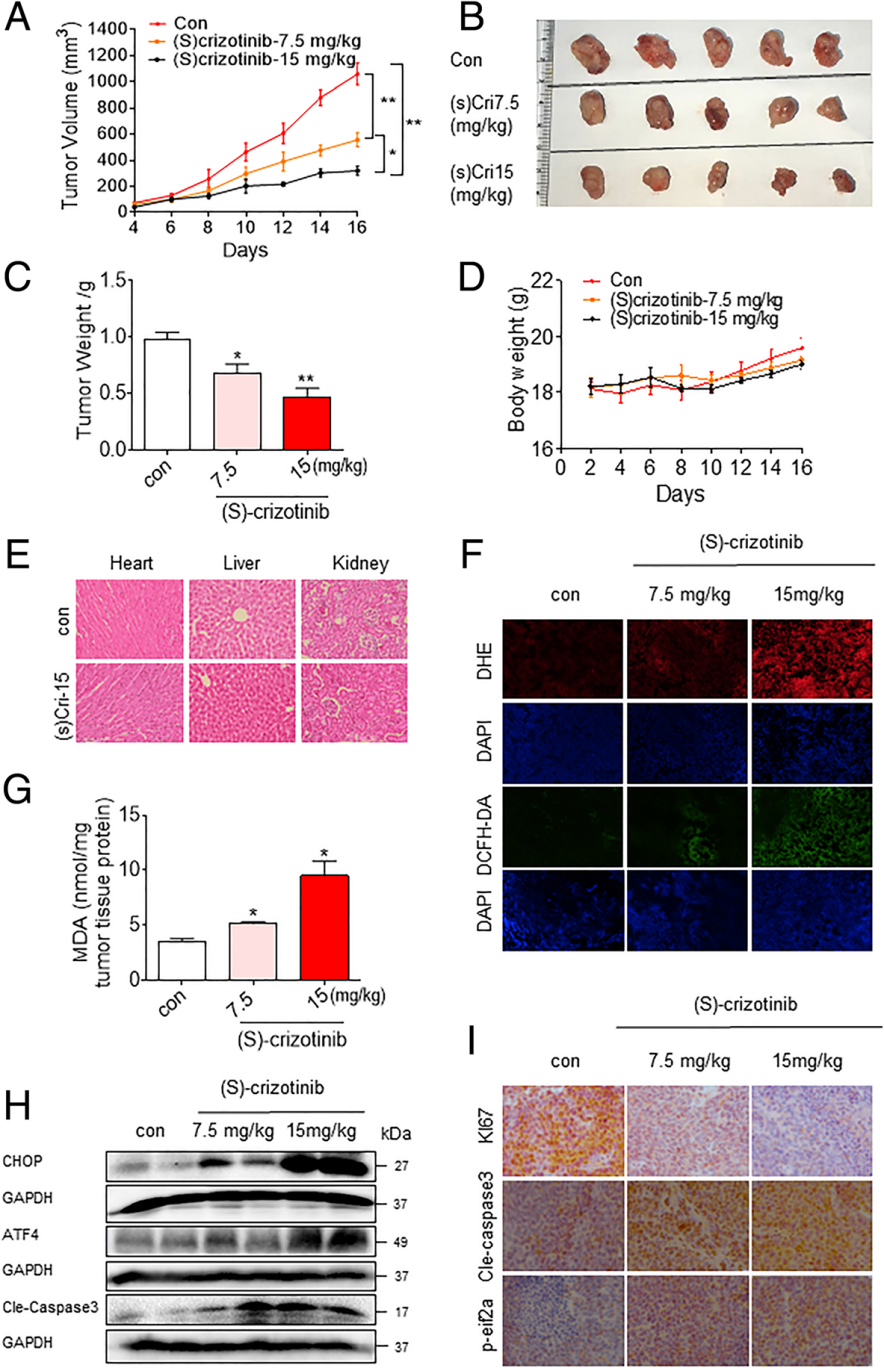
(S)-crizotinib inhibits NCI-H460 xenograft growth in vivo. **a** Tumor volume in vehicle and (S)-crizotinib treated mice. NCI-H460 cells were injected in the flanks of nude mice and tumors were allowed to develop for 6 d (50–100 mm^3^). Mice bearing NCI-H460 xenografts then received (S)-crizotinib at 7.5 or 15 mg/kg interperitoneally. Tumor volumes were calculated as described in the methods section. [*P < 0.05 and **P < 0.01]. **b** Images of resected tumor tissues at day 16. **c** Tumor weights at the end of the treatment period (16 d) [*P < 0.05, **P < 0.01 compared to vehicle control]. **d** Body weight of the mice in vehicle- and (S)-crizotinib-treatment groups. **e** H&E staining of heart, liver, and kidney specimens from mice harboring NCI-H460 tumor [magnification 20×]. **f** Fluorescence images of tumor specimens stained with DHE (red, upper panel) and DCFH-DA (green, lower panel). Tissue sections were counterstained with DAPI (blue). **g** Levels of MDA in tumor lysates [*p < 0.05 compared to vehicle control]. **h** Western blot analysis of ATF4, CHOP, and cleaved caspase-3 levels in resected tumor specimens. GAPDH was used as loading control. **i** Immunohistochemical staining of tumor sections for cell proliferation marker (Ki-67), apoptosis marker (cleaved caspase-3), and ER stress marker phosphorylated eIF2a. Representative images are shown

Our culture studies showed that (S)-crizotinib induces apoptosis in NSCLC cells by increasing ROS and activating ER stress and that these activities are independent of MTH1 alteration. We wanted to confirm these promising activities of (S)-crizotinib in vivo. To do this, we produced NCI-H460 xenografts in nude mice. We then treated mice harboring NCI-H460 tumors with (S)-crizotinib. Our studies show that treatment with (S)-crizotinib at 7.5 or 15 mg/kg for 10 days resulted in significant reductions in both tumor volume and tumor weight (Fig. 5a-c). No significant changes in body weights were observed in (S)-crizotinib-treated mice (Fig 5d). H&E staining analyses of vital organs (Heart, liver and kidney) further revealed that (S)-crizotinib treatment had nontoxicity (Fig 5e).

To determine if the same mechanisms are at play in vivo as observed in our in vitro studies, we assessed oxidative stress and cell growth in harvested tumor tissues. We stained the tumor tissues with ROS-sensitive dihydroethidium (DHE) and DCFH-DA. DHE forms a red fluorescent product upon reaction with ROS and intercalates with DNA. Microscopic examination revealed dramatically increased DHE and DCFH-DA fluorescence signals in tumor tissues from mice treated with (S)-crizotinib (Fig. 5f). MDA level is a useful quantitative indicator of cellular oxidative stress [28]. We found that (S)-crizotinib treatment also increased the levels of lipid peroxidation product (malondialdehyde; MDA) in tumor tissues (Fig. 5g), indicating increased ROS production. Together, MDA and DHE/DCF fluorescence indicate increased ROS production and oxidative stress in NCI-H460 tumors following (S)-crizotinib treatment. Western blot analyses and immunohistochemistry of the harvested tumor tissues also revealed that (S)-crizotinib treatment increased the levels of ATF4, CHOP, p-eIF2a (Fig. 5h and i) indicating activation of the ER stress pathway. In addition, we observed significant changes to cell growth parameters. Apoptosis, as assessed by cleaved caspase 3 levels, was increased in tumor cells following treatment with (S)-crizotinib and cell proliferation (ki-67 levels) was decreased (Fig. 5h and i). These data show that (S)-crizotinib exhibits potent anti-tumor activity in vivo by elevating ROS generation and inducing ER stress-related cell apoptosis.

## Discussion

Crizotinib is probably one of the most successful anticancer drugs of the past decade. Crizotinib shows high tumor response rate and prolonged progression-free survival compared to traditional chemotherapy [3, 29–31]. Crizotinib mediates these beneficial activities through targeting c-Met [7], CD74-ROS1 [8], and EML4-ALK [6]. In addition, recent reports indicate that crizotinib targets the nudix phosphohydrolase superfamily member, MTH1 [10]. In the latter case, (S)-crizotinib is found to be more potent than (R)-crizotinib [32]. Importantly, inhibiting MTH1 function by RNAi-mediated knockdown [10] or by (S)-crizotinib treatment [33] led to selective cell death in cancer cells but not normal counterparts. A number of MTH1 inhibitors have been reported to exert anti-proliferation effects, including (S)-crizotinb [33]. Interestingly, a recent study aimed at identifying potent MTH1 inhibitors showed that two compounds, NPD7155 and NPD9948, which inhibited MTH1 failed to show appreciable toxicity in cervical cancer HeLa cells [11]. Similarly, a series of MTH1 inhibitors were developed and tested on various cells alongside (S)-crizotinib [12]. Results of this study also showed limited effect of (S)-crizotinib on viability of a panel of cells. The investigators concluded that the reported effects of (S)-crizotinib may be ‘off-target’ effects, or in other words, independent of MTH1 inhibition. Therefore, the underlying molecular mechanism which contributes to (S)-crizotinib-induced death of human NSCLC cells remains enigmatic.

We set out to identify the mechanism of (S)-crizotinib-induced cell death in NSCLC. We hypothesized that (S)-crizotinib may exhibit antiproliferative activity in NSCLC cells independent of MTH1. We also postulated that the mechanism may involve generation of ROS, based on a few observations. First, crizotinib has been shown to increase oxidative stress in ovarian cancer cells [24]. Second, it is well established that cancer cells have higher basal levels of endogenous ROS compared to normal cell counterparts. ROS in cancer cells promotes cell proliferation and survival and accelerates mutations and genomic instability [16, 17]. The adverse effects of ROS such as promotion of cell death [18] is kept in check in cancer cells through high levels of antioxidant activities [34]. It is, therefore, possible that (S)-crizotinib increases ROS and unmasks the adverse, anti-proliferative effects of ROS in NSCLC cells. Indeed, our results show that (S)-crizotinib induces ROS generation in NSCLC cells within 3 h of exposure. This induction is mediated by mechanisms which are independent of MTH1 alteration or inhibition as knockdown of MTH1 with or without the (S)-crizotinib had no alter of ROS levels. Overproduction of ROS may lead to the induction of apoptosis and cell senescence through DNA damage [35, 36]. Incorporation of oxidized nucleotides into genomic DNA plays a crucial role in ROS-induced apoptosis [37]. Therefore, inhibition of MTH1 by (S)-crizotinib may amplify ROS toxicity by rendering cells unable to clear and sanitize oxidized nucleotides. We know that ROS is the central player as allowing cells to brace for the oxidative injury through pretreatment with NAC (as well as GSH) prevents (S)-crizotinib induced apoptosis in NSCLC cells.

In response to oxidative stress generated through elevation of ROS, (S)-crizotinib treatment activated the ER stress response [38]. ER stress manifests as increased phosphorylation of eIF2α which results in global repression of protein translation. One exception is ATF4 which is required for CHOP induction [39]. CHOP is considered as a marker of ‘commitment’ in the ER stress-induced apoptotic pathway [40, 41]. We have shown that (S)-crizotinib increases ROS levels and activates the ER stress pathway in NSCLC cells. However, when we knocked down the expression of CHOP, we noted significant but not complete normalization of cell death. However, NAC was able to completely attenuate (S)-crizotinib-induced cell death. These results suggest that ER stress pathway activation is a part of the mechanism but not the only path in cell death observed. The central player remains ROS but the mechanisms downstream may be multifactorial. It is possible that part of the mechanism may involve mitochondrial dysfunction. In a number of human cancers, we have shown that induction of ROS mediates apoptosis partly through producing mitochondrial deficits [42–45]. Whether (S)-crizotinib also causes mitochondrial alterations in NSCLC cells remains to be determined.

## Conclusions

In this study, we here investigated the potential usefulness and novel molecular mechanism of (S)-crizotinib in the treatment of NSCLCs. In our in vitro and in vivo data, we show that (S)-crizotinib treatment resulted in significant increases in ROS levels, and accumulation of ROS caused the activation of the ER stress apoptotic pathway. Moreover, changes in cell survival and ROS production triggered by (S)-crizotinib were carried out independently of its inhibitory effect on MTH1. We demonstrated that (S)-crizotinib remains a promising candidate for the treatment of NSCLCs and acts as a ROS regulator. Furthermore, ROS-mediated ER stress apoptotic pathway may be an unrecognized mechanism underlying the biological activity of (S)-crizotinib. Despite the advancement made in this study, the direct molecular target of (S)-crizotinib and mechanisms inducing the levels of ROS remain unclear. Further studies are necessary to establish this novel concept.

## Additional file

Additional file 1: Figure S1. (S)-crizotinib induced cytotoxicity in NSCLC cells. **Figure S2.** Effect of SOD on (S)-crizotinib-induced apoptosis in NSCLC cells. (DOC 548 kb)

## Abbreviations

ATF4: Activating transcription factor 4
Bax: Bcl-2 associated protein x
Bcl-2: B-cell lymphoma 2
CHOP: CAAT/enhancer binding protein (C/EBP) homologous protein
DAF-FM DA: 3-Amino,4-aminomethyl-2′,7′-difluorescein diacetate
DCFH-DA: 2′, 7′-dichlorodihydrofluorescin diacetate
DHE: Dihydroethdium
eIF2: Eukaryotic initiation factor 2
ER: Endoplasmic reticulum
FITC: Fluorescein isothiocyanate
GSH: Glutathione
Ki-67: Nuclear protein associated with cell proliferation
MDA: Malondialdehyde
MTT: 3-(4,5-dimethylthiazol-2-yl)-2,5-diphenyltetrazolium bromide
NAC: N-acetyl cysteine
PI: Propidium Iodide
ROS: Reactive oxygen species
SOD: Superoxide dismutase
