# Supplementary material for: (S)-crizotinib induces apoptosis in human non-small cell lung cancer cells by activating ROS independent of MTH1
Source: J Exp Clin Cancer Res. 2017 Sep 7;36:120. doi: 10.1186/s13046-017-0584-3 (PMC5590185; doi:10.1186/s13046-017-0584-3)
Supplement: Additional file 1: Figure S1. — (S)-crizotinib induced cytotoxicity in NSCLC cells. Figure S2. Effect of SOD on (S)-crizotinib-induced apoptosis in NSCLC cells. (DOC 548 kb) [file 13046_2017_584_MOESM1_ESM.doc]

***Supporting information***

**(S)-crizotinib induces apoptosis in human non-small cell lung cancer cells by activating ROS independent of MTH1**

Xuanxuan Dai^1,2^, Guilong Guo^2^, Peng Zou^1^, Ri Cui^1^, Weiqian Chen^3^, Xi Chen^1^, Changtian Yin^2^, Wei He^1^, Rajamanickam Vinothkumar^1^, Fan Yang^2^, Xiaohua Zhang^2,*^, Guang Liang^1,*^

1. Chemical Biology Research Center, School of Pharmaceutical Sciences, Wenzhou Medical University, Wenzhou, Zhejiang 325035, China

2. Department of Surgical Oncology, The First Affiliated Hospital of Wenzhou Medical University, Wenzhou, Zhejiang 325035, China

3. Department of Interventional Radiology, The Fifth Affiliated Hospital of Wenzhou Medical University, Lishui, Zhejiang 323000, China

**Contents**

Supplemental information includes 2 figures.


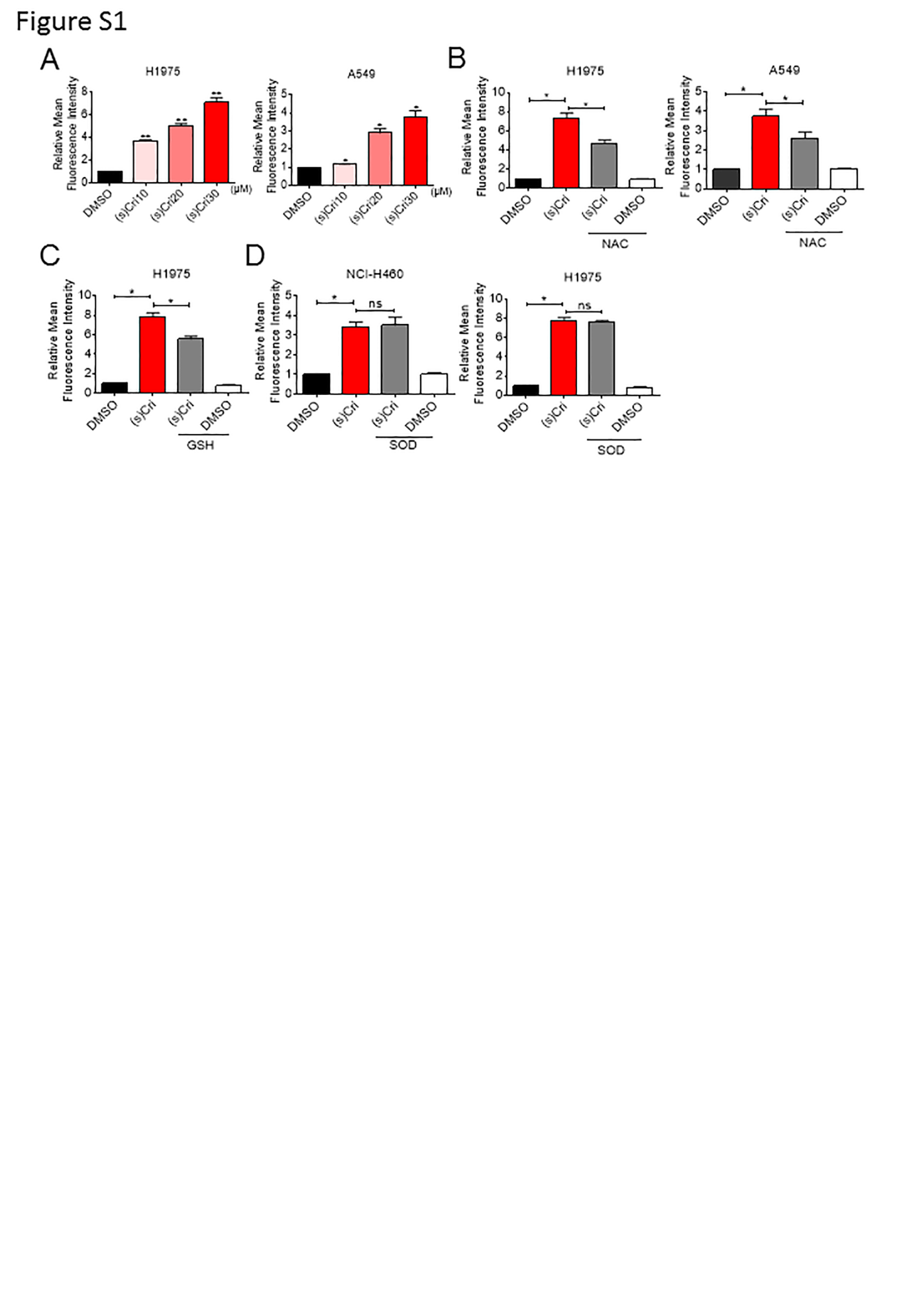


**Figure S1. (S)-crizotinib induced cytotoxicity in NSCLC cells. a** Intracellular ROS induced by increasing doses of (S)-crizotinib in H1975 and A549 as assessed by DCFH-DA staining. Mean fluorescence intensities were calculated and represented as the percent of control [*p < 0.05, **p < 0.01 compared to DMSO control]. **b-d** NSCLC cells were treated with 30 µM (S)-crizotinib with or without 1 h pretreatment with 5 mM NAC **(b)**, 5 mM GSH **(c)**, or 300 U/mL SOD **(d)**. The relative mean fluorescence intensity are shown [*p < 0.05, ns = no significance].


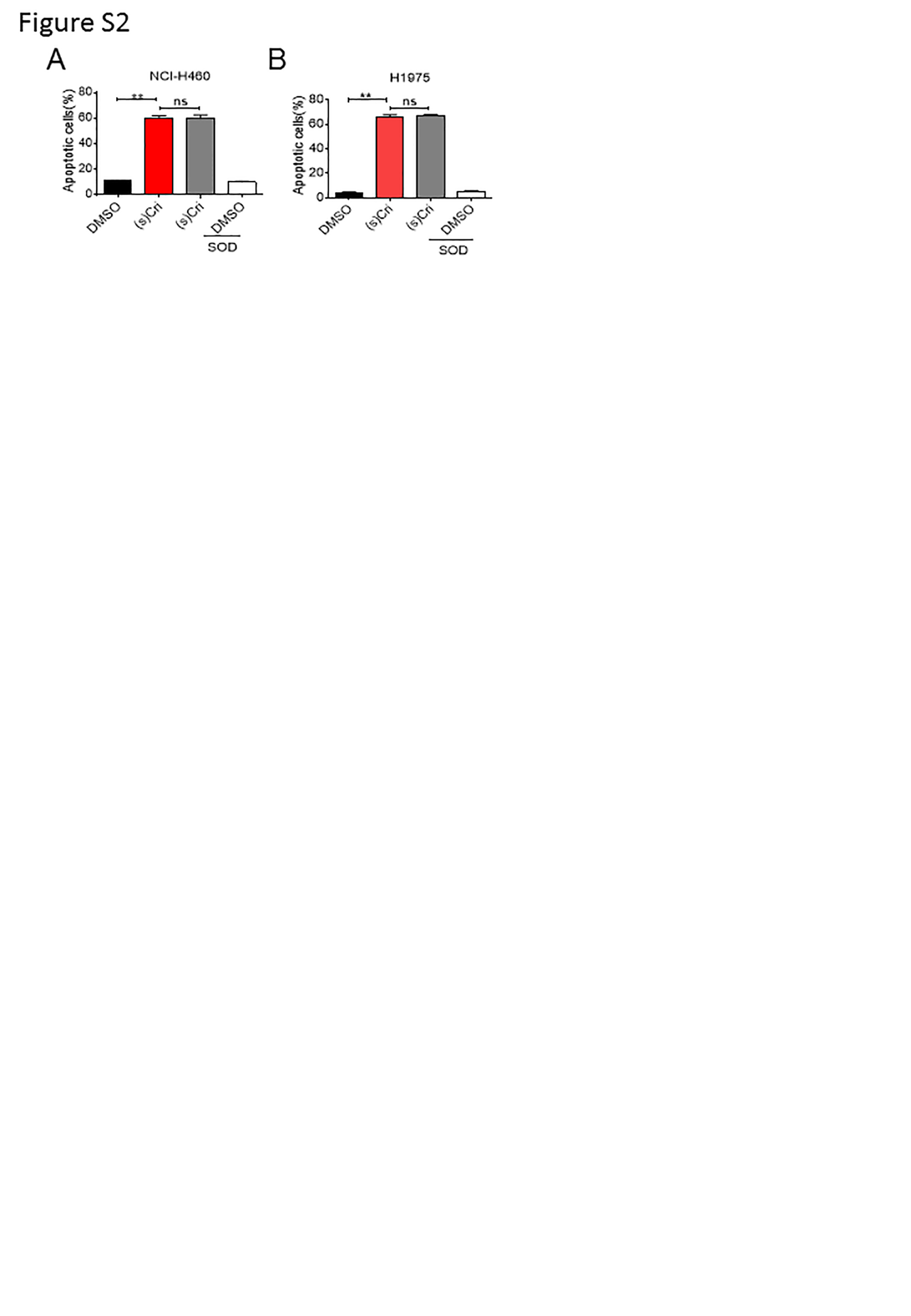


**Figure S2. Effect of SOD on (S)-crizotinib-induced apoptosis in NSCLC cells.** NSCLC cells (NCI-H460, **a**; H1975, **b**) were pretreated with 300 U/mL SOD for 1 h before exposure to 30 µM (S)-crizotinib for 24 h. Apoptosis was then measured by annexin V/PI staining. Percentage of apoptotic cells was calculated [**p < 0.01, ns = no significance].
